# Genetic and Molecular Genetic Basis of Nuclear-Plastid Incompatibilities

**DOI:** 10.3390/plants9010023

**Published:** 2019-12-23

**Authors:** Vera S. Bogdanova

**Affiliations:** Institute of Cytology and Genetics, Siberian Branch of Russian Academy of Sciences, Novosibirsk 630090, Russia; vera@bionet.nsc.ru

**Keywords:** cytonuclear incompatibility, genetic analysis, plastids

## Abstract

Genetic analysis of nuclear-cytoplasm incompatibilities is not straightforward and requires an elaborated experimental design. A number of species have been genetically studied, but notable advances in genetic mapping of nuclear loci involved in nuclear-plastid incompatibility have been achieved only in wheat and pea. This review focuses on the study of the genetic background underlying nuclear-plastid incompatibilities, including cases where the molecular genetic basis of such incompatibility has been unveiled, such as in tobacco, *Oenothera*, pea, and wheat.

## 1. Introduction

Nuclear-cytoplasmic incompatibility, also referred to as cytonuclear incompatibility, is generally accepted to be an important evolutionary factor [1] that contributes to the establishment of reproductive isolation [2]. Apart from its fundamental evolutionary role, reproductive isolation of diverged plant species and subspecies has a strong impact on breeders’ efforts, since more or less, distant wild relatives of cultivated plants are frequently recruited to improve the existing crops [3,4]. This approach to plant breeding and related studies have mostly been directed to improve crop yield, resistance to various pathogens, or adaptation to unfavorable climatic changes [5]. The development of molecular methods has favored introgression of certain genes, extended genetic blocks from wild species, or traditional landraces into cultivars [6,7]. The first experience of wide hybridization of crops with their wild relatives faced problems of hybrid incompatibility, including hybrid sterility, hybrid breakdown, and genetic linkage of loci responsible for desirable and undesirable traits [5]. In some plants, the genetic basis of hybrid incompatibility has been studied (reviewed in [8,9,10,11]). A large number of cases have been reported where the decreased fitness of hybrids was related to impaired functions of mitochondria or plastids [8,12,13]. Here, we review the cases where genetic analyses have been undertaken to uncover the genetic background of incompatibility of the nucleus with plastids and its molecular genetic mechanisms. 

## 2. Occurrence of Nuclear-Plastid Incompatibility

A comprehensive list of the registered cases of nuclear-plastid incompatibilities is presented in [12] and includes representatives of 14 plant genera. However, in most cases, studying nuclear-plastid incompatibility results in a mere affirmation that incompatibility exists, with systematic studies carried out for only a limited number of genera. For example, nuclear-chloroplast incompatibility was genetically studied in interspecific hybrids of *Oenothera* L. [14,15,16,17,18,19,20,21], *Zantedeschia* Spreng. [22,23], and *Passiflora* L. [24]. 

Nuclear-plastid conflict was thoroughly described in a somewhat artificial system, intergeneric cybrids (somatic cell hybrids) of Solanaceae [25,26,27,28,29,30,31,32,33,34]. In this case molecular basis of the conflict has been dissected (see below).

In *Pisum* L., some intraspecific crosses involving representatives of cultivated and wild subspecies have resulted in hybrids with abnormal leaf development, variegated chlorosis [35], and abnormalities in meiosis, resulting in partial fertility of pollen and ovuli [36] which was attributed to nuclear-plastid incompatibility [37,38]. This case has been subjected to extensive genetic and molecular genetic analysis.

Nuclear-plastid incompatibilities were also revealed in the hybrids resulting from crosses of phylogeographic lineages within a species of the herbs *Silene nutans* L. [39] and *Campanulastrum americanum* (L.) Small [1,40].

In *Triticum* L., cytoplasmic effects in the course of backcrossing have been studied for a long time [41]. One of the most frequent manifestations of the alien cytoplasm was male sterility [42,43], while other effects included variegated leaf pigmentation [44], pistillody, germless grain formation, haploid and tween seedling formation, depressed growth vigor, premature sprouting, delayed heading [42], and decreased plant stature [45,46]. It was not always clear which cellular compartment was responsible for such an effect, mitochondria, plastids, or both [47,48,49,50]. This case deserves attention due to the thorough genetic and molecular genetic analyses performed in the study.

## 3. Genetic Analysis of Nuclear-Plastid Incompatibility

### 3.1. Genetic Analysis of Nuclear-Plastid Incompatibility in Barrel Medic

Asymmetry in the phenotypes of progeny was observed in hybrids obtained in reciprocal crosses of two Israeli local forms of *Medicago truncatula* Gaertn. When the dusky green race (Du) originating from Jawniel was pollinated by the bright green race (Br) from Mt. Tabor, the hybrids had pale or very pale cotyledons, with leaves that manifested all levels of subnormal green pigmentation, from almost colorless to normal green. Plants with pale and green sectors were met. Seed fertility was low, with two to three seeds per pod as compared with eight to nine seeds in parental lines. The reciprocal F_1_ hybrids had light-green cotyledons. New leaves were pale but during the growth acquired healthy green coloration, but the seed set was poor, with two to three seeds per pod. In the hybrids, where dusty green race was maternal, chlorophyll content was 2.5 times lower than in reciprocals, and it was lower in both types of hybrids as compared with both parental lines [51]. The results of segregation in F_2_ were confusing, no clear ratios of phenotypes varying in a wide range of chlorophyll abnormalities could be traced. The variability of the segregation ratios is explained by taking into account biparental mode of chloroplast inheritance in *M. truncatula* which was shown later [52]. Test cross Br × (Du × Br) produced two types of progeny, with green cotyledons and green leaves (22 plants) and with light-green cotyledons and pale leaves (31 plants) [51], which does not contradicts the 1:1 ratio, and therefore can be interpreted as monogenic segregation. In the reciprocal cross (Du × Br) × Br, the progenies suffered from a more severe plastid dysfunction; they were scarce, producing in total 17 plants, of which 10 had light-green leaves and the rest had pale leaves [51]. This also does not contradict the 1:1 ratio and can be interpreted as segregation of alleles of one nuclear gene. However, until the origin of the plastids in the crosses is clearly established, the study’s [51] inferences should be considered with care.

Another plastid disorder observed in these crosses manifested as a fine mosaic of green, pale, or yellowish tissue variegation. In crosses with the Br line, green and variegated F_2_ progenies segregated at a ratio close to 3:1 which indicated involvement of one nuclear factor [53]. However, this analysis was done only in the background of the presumable Br cytoplasm, and therefore the relevance of this nuclear factor to cytonuclear compatibility remains unclear. 

### 3.2. Genetic Analysis of Nuclear-Plastid Incompatibility in Tan Wattles

Crosses were made of two species of tan wattles, green (*Acacia decurrens* Willd.) and black (*A. mollissima* Willd.) with a goal to produce hybrids of economic value. The hybrids were of little commercial importance but produced some data on the occurrence of chlorotics in the offsprings [54]. Upon cross-pollination in both reciprocal directions, F_1_ hybrids were formed that had low fertility and various degree of chlorosis. During growth, normal bluish-green coloration was restored which hampered classification of chlorotic versus normal phenotype. In the F_2_ generation, chlorophyll pigmentation varied widely, from white and very pale green to green and bluish-green. Clear Mendelian ratios were not observed, possibly due to uncertainties in phenotypic classification. Thus, the data on F_2_ segregation did not contribute to drawing conclusions on the genetic basis of the hybrid chlorosis. Therefore, backcrosses were made, where F_1_ hybrids with the cytoplasm from green wattle were pollinated with green or black wattle. After pollination with black wattle, the majority of progenies had normal chlorophyll pigmentation, although some chlorotics could be observed. Unexpectantly, after pollination with green wattle, phenotypic segregation was observed, resembling that in F_2_. The proportion of mosaics was high at about 37%, in spite of the same origin of the cytoplasm and pollen. It was concluded that the genome of green wattle contained some genetic factor(s) that caused instability of chlorophyll pigmentation (appearance of mosaics) as a result of improper interaction of nuclear alleles from green and black wattles [55]. This conclusion, again, should be considered with care until the source of cellular organelles is established with certainty. 

### 3.3. Genetic Analysis of Nuclear-Plastid Incompatibility in Evening Primroses

In *Oenothera*, crosses were made to study the genetic bases of hybrid chlorosis occurring in incompatible combinations of certain types of nuclear and plastid genomes. Nuclear genomes met in related species of *Oenothera* were classified into three types, A, B, and C, while plastid genomes (plastomes) were classified into five types, I–V [14]. Various combinations of the genome variant and plastomes were found to have different degrees of compatibility. 

In *Oenothera atrovirens* Shull & Bartlett (genome C, plastome IV), the nuclear gene *Fl* determines stem shape, while two *lor* loci affect the leaf width. Brought into other cytoplasm type by crosses, *lor lor* plants displayed strong chlorophyll deficiency; *Fl Fl* plants were weak and pale, and the combination *lor lor Fl Fl* caused embryonic lethality. In the presence of the native *atrovirense* plastids (situation of heteroplasmy), such homozygotes were pale brownish plants and died early [56]. In *Oenothera*, homologous recombination and free segregation of chromosomes due to permanent translocation heterozygosity are generally suppressed [57], thus, the *Fl* and *lor* visible alleles marked the chromosome arms carrying loci of cytonuclear compatibility.

Hybridization between species with genome A and plastome I, *Oenothera hookeri* Torr. et A. Gray or *O. elata* Kunth and species *O. argillicola* Mack. with genome C and plastome V, resulted in formation of heterozygotes for the genomes A and C and plastids from both parents (as typical for *Oenothera*). Such hybrids from both reciprocal crosses suffered from severe chlorosis and died at the seedling stage due to incompatibility of the genome A with the plastome V and of the genome C with the plastome I. For this reason, it was impossible to obtain F_2_ from such crosses. This difficulty was overcome by developing an intermediate, a substitution line of *O. argillicola* which carried plastome IV instead of the plastome V [58], characterized by wide compatibility. When this line was crossed with *O. elata*, the resulting hybrids were heterozygous for the genomes A and C and carried plastids from both parents. Plant sectors carrying plastome IV were green, whereas sectors carrying plastome I were chlorotic but formed flowers that were used for subsequent test crosses. In such a manner, progeny was obtained of the backcross A, C × A, A in the background of the plastome I [59]. The progenies were classified into normal or chlorotic, and cytological analysis of meiocytes was employed to determine which of the chromosomes from the complement C were associated with the chlorotic phenotype. In the heterozygote for genomes A and C, reciprocal translocations resulted in formation of six-chromosome rings, four-chromosome rings, and free bivalents in prophase of meiosis. These were passed to the progeny in different combinations that could be determined cytologically. It was concluded that the presence of individual foreign chromosomes did not cause chlorosis, while their various combinations brought about different degrees of chlorosis. The stronger degree of chlorosis was associated with a higher number of foreign chromosomes in a hybrid genome, that is, the responsible nuclear factors were scattered all over the genome. Similar results were obtained in another cross designed to estimate the effect of the chromosome complement A on the compatibility with the plastome V, A, C × C, C in the background of plastome V [59].

Genetic analysis was carried out for the incompatibility of genome A with plastome III, which was manifested as chlorophyll deficiency normalized in the course of plant growth (*virescent* phenotype), which made it possible to obtain and study segregation ratios in the progeny. A cross was made between *O. elata* (genome A) and *O. grandiflora* L. Hér. ex Aiton. (genome B), and both parental lines carried the same plastome III transferred by repeated crosses from *O. glazioviana* Micheli. Among the F_2_ plants, some had normal green pigmentation, while others had chlorophyll deficiency of varying degrees. Unfortunately, the exact ratio of green and non-green plants could not be established due to the difficulties of classification, but it was clear that at least two nuclear factors segregated in the F_2_ population which generated incompatible nuclear-plastid combinations. Analysis of a large number of molecular markers placed one of these factors onto the genetic map of the chromosome 4 and the other-onto the chromosome 7 [60]. 

### 3.4. Genetic Analysis of Nuclear-Plastid Incompatibility in Calla Lilies

In an ornamental plant *Zantedeschia*, nuclear-cytoplasmic conflict occurs upon crossing of an evergreen species *Z. aethiopica* (L.) Spreng. (AE) with representatives of the winter-dormant species group, in particular, *Z. odorata* P.L. Perry (OD). In F_1_ hybrids from reciprocal crosses, OD plastids were associated with albino phenotype, and AE plastids brought about virescent plants [22]. To study the genetic basis of the incompatibility, segregation ratios were analyzed in F_2_ and in backcrosses. In the F_2_ population resulting from the cross AE × OD, green, virescent, and albino plants appeared, although in F_1_, all plants were virescent, which was evidence that segregation of nuclear genes was involved into the incompatibility. It was difficult to distinguish between green and virescent plants, while albino plants, which could only be maintained via embryo culture [61], comprised 18 of 58 germinated embryos. Green plants were rare, and the authors concluded that the nuclear-plastid conflict in *Zantedeschia* was mediated by at least two nuclear genes [23], although the proposed model did not explain the proportion of albino plants. At the same time, the hypothesis of one nuclear gene cannot be discarded. The ratio 18:58 in F_2_ does not contradict 1:4 expected for segregation of one recessive allele. However, the segregation ratio in backcrosses was contradictory; there appeared a large number of albino plants in the background of the own cytoplasm. This could be attributed to nuclear–nuclear interaction, but the results should be treated with care because of possible paternal leakage of chloroplasts. 

### 3.5. Genetic Analysis of Nuclear-Plastid Incompatibility in Azaleas

In intersubgeneric crosses of evergreen (EV) azaleas of the genus *Rhododendron* L. with yellow-flowered deciduous azalea *R. japonicum* (A. Gray) Kron f. *flavum* (JP), unilateral genome-plastome incompatibility occurred in F_1_ progenies when EV plants were used as seed parent. The majority of the progenies suffered from albinism because of incompatibility between EV-plastids and JP nuclear genome, and green plants occurred only in the case of occasional plastid DNA inheritance from the JP-parent [62]. Crossing of diploids, 2 × EV (genome constitution EE) × 2 × JP (genome constitution JJ) produced albinas with genomic constitution EJ. Crosses were made of azaleas of different ploidy, 4 × EV × 4 × JP and 2 × EV × 4 × JP, that gave progenies with genomic constitution EEJJ and EJJ, respectively. All of the progenies manifested nuclear-plastid incompatibility. This indicated that the JP nuclear genome carried dominant factor(s) that restricted development of EV chloroplasts. At the same time, plants with genomic constitution EEJ, resulting from the cross 4 × EV × 2× JP, were green, although they carried maternally-inherited EV plastids, and were not rescued by occasional leakage of paternal chloroplasts. Thus, incompatibility of EV plastids with JP genome could be compensated by increasing the EV dosage in the nuclear genome in the offspring [63].

### 3.6. Genetic Analysis of Nuclear-Plastid Incompatibility in Cereals

In Triticeae, nuclear-cytoplasmic conflict (apart from cytoplasmic male sterility) has been known for a long time, although it was not always clear which cytoplasmic component was responsible for the incompatibility [41]. However, here, we discuss this case in detail because it has been profoundly genetically studied. 

Incompatibility of some *Triticum* species with the cytoplasm of *Ae. tauschii* Coss. [45] (the authors used the name *Ae*. *squarrosa* L.) and *Ae. longissima* Schweinf. et Muschl. [64] has been genetically studied. 

A series of alloplasmic lines was obtained which combined *Ae. taushii* cytoplasm with nuclear genomes of different representatives of Dinkel wheat (genome composition AABBDD), Emmer group (genome composition AABB), and Timopheevii group (genome composition AAGG) [45]. After six generations of backcrossing with the pollen of hexaploid wheats (AABBDD), fertile alloplasmic lines were obtained with a normal chromosome configuration (21 bivalents) void of observable morphologic or physiologic irregularities. When wheats of the Emmer group (AABB) were used as the recurrent parent, all alloplasmic lines with the *Ae. tauschii* cytoplasm carried one additional chromosome. This indicates that some genetic factor(s) located in the additional chromosome were required for compatibility of nuclear genomes with the cytoplasm. Sporophytes and male gametophytes without the additional chromosome died. An analysis of a set of Chinese Spring alloplasmic mono-trisomic lines, in which one of the chromosomes of the complement D was substituted by a homoeologous chromosome from the A or B complement, led to a conclusion that factor(s) responsible for the compatibility of the nucleus and the cytoplasm were located on the chromosome 1D. Equivalent nuclear factor(s) providing compatibility with the *Ae*. *tauschii* cytoplasm were also present in the genome of tetraploid wheats of the Timopheevii group (AAGG) [45].

The additional 1D chromosome providing compatibility of tetraploid genomes of the AABB type with the cytoplasm of *Ae*. *tauschii* can be transmitted or not transmitted via female gametes (not following Mendelian ratios). As a result, when alloplasmic lines were pollinated by the recurrent parent lacking the additional chromosome, the resulting seeds segregated into viable (carrying 1D chromosome, 2n = 29, 3n = 44) and abortive (lacking 1D chromosome, 2n = 28, 3n = 42). In some cases, viable but shriveled seeds formed that produced miniature plants which developed severe chlorophyll variegation under low temperatures. The lines which brought about formation of abortive seeds, when used as pollen parents, were referred to as type I and classified as incompatible with the cytoplasm of *Ae. tauschii*. The lines which brought about shriveled but viable seeds were classified as partially compatible with the cytoplasm of *Ae. tauschii* and referred to as type III. Type II, which was fully compatible with the cytoplasm of *Ae. tauschii*, was represented by the wheats from the Timopheevii group. 

The existence of the miniature plants that survived without the additional chromosome allowed for performing genetic analysis. The miniature and weak plants with or without chlorophyll variegation appeared in the progeny of crosses with partially compatible lines. Analysis of the number of progenies in phenotypic classes according to chlorophyll variegation and plant vigor allowed the authors to propose segregation of two nuclear genes. They concluded that one of the nuclear genes, denoted as *Cp*, was involved into an interaction with chloroplasts; it had allelic state *Cp1* in incompatible lines and allelic state *Cp2* in partially compatible lines. The homozygotes *Cp1 Cp1* died resulting in abortive seeds, the heterozygotes *Cp1 Cp2* were manifested as shriveled seeds that grew into variegated plants, and the homozygotes *Cp2 Cp2* had normal chlorophyll pigmentation. The second nuclear gene was supposed to be related to plant vigor and was denoted as *Cv*. It had allelic state *Cv1* in incompatible lines and allelic state *Cv2* in partially compatible lines. The homozygotes *Cv1 Cv1* were miniature, the heterozygotes *Cv1 Cv2* were weak plants if they lacked chlorophyll variegation (in homozygotes *Cp2 Cp2*) or miniature plants if they suffered chlorophyll variegation (heterozygotes *Cp1 Cp2*), and the homozygotes *Cv2 Cv2* were of subnormal plant vigor [45]. 

Genetic analysis was performed on the factor(s) conferring compatibility of the *T. timopheevii* (Zhuk.) Zhuk. genome with the cytoplasm of *Ae. tauschii* which was denoted as *Ncc-tmp* (from nucleus-cytoplasm compatibility-*timopheevii*) and possibly corresponded to the *Cp3* allele (described by Ohtsuka [45,65]. This factor was introgressed into *T. durum* Desf. cv. Langdon by a series of backcrosses with the pollen of cv. Langdon in the background of the *Ae. tauschii* cytoplasm. Since the additional chromosome 1D was absent, the introgressed line retained the *Ncc-tmp* factor indispensable for formation of viable seeds. 

The RAPD (random amplified polymorphic DNA) markers were developed which were linked to the compatibility factor *Ncc-tmp* proceeding from *T. timopheevii* Zhuk. [65]. Southern blotting was used to determine which of the chromosomes carried *Ncc-tmp* in a series of disomic lines where one of the chromosomes of the A or B complement was substituted by the homoeologous D chromosome. It was shown that a specific signal disappeared from the genome of *T. durum* cv. Langdon along with the substitution of the 1A chromosome with 1D. Thus, *Ncc-tmp* factor was associated with the chromosome 1A and denoted *Ncc-tmp1A* [66]. 

A number of test crosses were made to determine whether additional compatibility factors were present in the G genome of *T. timopheevii* [66,67]. Hybrids were obtained of *T. timopheevii* with *T. durum* in the background of the cytoplasm of *Ae. tauschii*. After a test cross, segregation of the compatible and incompatible alleles was estimated by the ratio of plump and shriveled seeds. If the initial line input one dominant allele into the heterozygote used for the test cross, the expected segregation ratio was 1:1. If the initial line contributed two dominant compatible alleles, from A genome and G genome, the possible input of such heterozygote into the offspring could be one of the following four options: One dominant allele from genome A, one dominant allele from genome G, both dominant alleles, or neither of compatible alleles. The first three options, unlike the fourth, should result in formation of viable seeds, therefore, the expected segregation ratio was 3:1, although some deviations were possible since chromosomes from the G genome were not always transmitted. Segregation analysis in the progeny of a number of test crosses showed that the G genome of *T. timopheevii* contained a functional allele of nuclear-cytoplasm compatibility, which was denoted as *Ncc-tmp1G* [67]. 

Southern blot hybridization, of DNA clones mapped on homoeologous group I using RAPD markers and linked to *Ncc-tmp1A* as a probe, showed that this locus’s position is close to the centromere of the chromosome 1A [67]. Such a location of compatibility factors appears to be conservative in the cereals. For example, a DNA fragment from the centromeric region of the rye 1R chromosome seemed to contain genetic factor, conferring compatibility of the genome of the bread wheat (cv. Chinese Spring) with rye cytoplasm *Secale cereale* L. in a substitution line [68]. Chromosomes from the homoeologous group 1 restored plant vigor and male fertility in alloplasmic wheat with *Agropyron* Gaertn. [69] and *Elymus* L. [70] cytoplasm.

Another series of experiments [71,72] performed genetic analysis of the factors conferring compatibility of the genomes of tetraploid wheats with the cytoplasm of *Aegilops longissima*. Similar to the case of *Ae. tauschii*, a compatible line of *T. turgidum* was obtained by introgression of the compatible allele of a locus denoted as *scs* (species cytoplasm specific) from *T. timopheevii.* In the presence of the introgressed allele, *scs ^ti^*, viable seeds were formed; however, male gametophytes were inviable [71]. Restoration of male fertility required introgression of the compatible allele of another locus denoted as *Vi* (vitality) [72]. The compatible *Vi* allele appeared to arise by a spontaneous mutation in an alloplasmic line of *T*. *aestivum* with the cytoplasm of *Ae. cylindrica* Host [73]. 

Segregation for *scs* and *Vi* alleles was analyzed with the use of RFLP-markers (restriction fragment length polymorphism). The *Vi* locus was positioned in the distal part of the chromosome arm 1BS, and *scs* locus, in the chromosome arm 1AL close to the centromere [64], almost at the same location as independently introgressed *Ncc-tmp1A* [66]. It is highly possible that *Ncc-tmp1A* conferring compatibility of the nuclear genome of *T. turgidum* with the cytoplasm of *Ae. tauschii*, and *scs ^ti^* conferring compatibility with the cytoplasm of *Ae. longissima*, correspond to the same locus [67]. 

A more precise mapping of the *scs ^ti^* was made in the cross of euplasmic lines with the cytoplasm of durum wheat. One of the parent lines was homozygous for the introgressed *scs ^ti^* allele from *T. timopheevii*. The resulting F_2_ progenies were genotyped by test crosses as pollen parents with a tester line having the cytoplasm from *Ae. longissima* and one copy of the allele *scs ^ti^*. F_2_ plants possessing two functional copies of *scs ^ti^* upon crossing gave only plump viable seeds; plants with one functional copy of *scs ^ti^* (hetero-, or possibly hemizygotes) segregated with the ratio of 3:1 of plump to shriveled unviable seeds, and plants lacking functional copy of *scs ^ti^* gave the segregation ratio of 1:1 of plump to shriveled seeds. In all plants, the allelic state of a number of RFLP, AFLP (amplified fragment length polymorphism), and microsatellite markers was scored. On the basis of the segregation data, a genetic map of the centromeric region of the 1AL chromosome arm was constructed. Microsatellite markers flanking the *scs* locus, *Xbcd12* and *Xbcd1449-1A.2,* were mapped at a distance of 2.3 and 0.6 cM, respectively [46]. Later, the mapping population was extended to comprise 2158 plants, additional molecular markers were developed, and a more detailed genetic map was constructed of the chromosome 1A region. The markers nearest to *scs ^ti^*, *Xwmc120* and *Xtc252572-2-a,* were mapped at a distance of 0.52 and 0.61 cM, respectively [74]. 

The genetic factor conferring compatibility of the 1D chromosome of hexaploid wheat with the cytoplasm of *Ae. longissima* was denoted as *scs ^ae^* and mapped using the radiation hybrid method in 1DL chromosome arm [75]. An alloplasmic line of *T. turgidum* was used, in which a part of 1A chromosome was substituted with an almost entire 1D chromosome from *T. aestivum* [76]. Under impact of radiation, chromosomes were disrupted, and some chromosome fragments were lost. To compensate for the loss of chromosome matter, plants grown from irradiated seeds were crossed with the euplasmic line of *T. turgidum* (genome constitution AABB), to obtain heterozygotes (so-called radiation hybrids), in which deleterious effect of the breaks of chromosomes from A and B genomes was compensated by the presence of intact chromosomes. But the additional 1D chromosome turned out to be hemizygous, so that the breaks in this chromosome were not compensated. The loss of some chromosome portions caused by irradiation was determined by analysis of molecular markers specific for 1D. Since *scs ^ae^* allele is indispensable for compatibility with the cytoplasm of *Ae. longissima* it was inevitably present in all viable plants. Eight molecular markers covering a stretch of about 8.3 Mb that were constantly present in radiation hybrids determined the position of the *scs* locus on the chromosome arm 1DL [75]. Later, additional markers were involved in radiation hybrid mapping. In the plants grown from the irradiated seeds, the allelic state of the *scs* locus was determined by crossing followed by segregation analysis of the seed phenotype (plump vs. shriveled). In addition, the presence or absence of the 1D-specific markers was scored. As a result, the approximate length of the 1D chromosome stretch containing *scs ^ae^* was shortened to 1.1 Mb. On the basis of the synteny of the genomes of wheat, rice, sorghum, and *Brachypodium* P.Beauv., 16 genes were predicted to be located in this region [77].

### 3.7. Genetic Analysis of Nuclear-Plastid Incompatibility in Peas

In pea, one accession of wild subspecies (VIR320) was found to be associated with a severe impairment of plastid function when used as a cytoplasm donor in crosses with the cultivated material [35,37,38]. The occurrence of the incompatibility at the intraspecies level facilitated hybridologic analysis. However, direct segregation ratio count was impossible, since F_1_ hybrids produced only a few seeds or none at all. 

To study the genetic basis of the nuclear-plastid incompatibility in pea, a mapping population of recombinant inbred lines (RIL) was established based on a cross of a tester line carrying a number of visible markers with the wild line VIR320 [78]. The allelic state of compatibility loci of the plants from the RIL population was determined by crosses in which pollen of the tested plants was applied to the pistils of VIR320 as a donor of poorly compatible plastids. Nuclear-cytoplasm combination was regarded as incompatible if all of its carriers were deficient in chlorophyll pigmentation, which could vary in color from white to pale green and had reduced leaf organs. Otherwise, the combination was deemed to be compatible.

It was expected that those lines that inherited alleles of the compatibility factor(s) from the tester line would produce offspring with typical signs of nuclear-plastid incompatibility, while those carrying alleles of compatibility factor(s) from VIR320 would give progenies without clear signs of the plastid dysfunction. Analysis of genetic linkage using a number of visible and molecular markers led to a conclusion that a typical syndrome of nuclear-plastid incompatibility was conditioned by two unlinked nuclear loci, denoted as *Scs1* and *Scs2,* by analogy with the earlier mentioned wheat gene. *Scs1* was mapped on linkage group III, and *Scs2* was mapped on linkage group V. If both *Scs* loci were heterozygous, severe chlorophyll deficiency, reduction of leaves and stipules, and low-fertile pollen (33% on average) developed in the background of the cytoplasm of the wild pea. If only one of these loci was heterozygous, the plants appeared normal, although somewhat paler than reciprocals [78].

Having established that nuclear-plastid incompatibility was conditioned by the two unlinked loci, it became possible to analyze inheritance of each of them separately, in the crosses not complicated by severe breakdown of the progeny. In a series of test crosses, it was shown that *Scs1* allele from cultivated pea was lethal for sporophytes and male gametophytes in the background of the wild pea cytoplasm. *Scs2* allele was not lethal, but was underrepresented among male gametophytes and sporohytes, indicating that some yet unknown genetic interactions brought about lethality to the carriers of this allele [79]. In a number of crosses, *Scs1* and *Scs2* loci were genetically mapped; *Scs1* in an interval of about 2.5 cM on linkage group III, and *Scs2* in an interval 2.0 to 15.1 cM varying from cross to cross on linkage group V [79]. 

### 3.8. General Consideration on Genetic Control of Nuclear-Plastid Incompatibility

Summary of studies made to uncover genetic basis of cytonuclear incompatibilities is given in the Table 1.

The reviewed genetic studies dealt with the dominant cases of nuclear-plastid incompatibility, that is, manifested in F_1_, although incompatible or poorly compatible paternal alleles of the nuclear loci were present in only one copy in the heterozygous state. In these cases, one copy of the compatible maternal allele was insufficient to maintain normal chloroplast functioning. One of possible explanations is that chloroplast genes, unlike nuclear ones, exist in multiple copies, with hundreds of nucleoids per plastid and dozens of plastids per cell [80]. Plastid gene products frequently participate in formation of multisubunit complexes together with nuclear gene products, but expression levels of plastid genes, measured as the number of corresponding mRNAs, can be more than ten times higher than that of nuclear counterparts [81]. For this reason, nuclear function in heterozygote could be insufficient for maintaining a proper stoichiometry of the gene products. Notably, increasing the ploidy of a seed parent abolishes the manifestation of incompatibility [63].

## 4. Molecular Genetic Analysis of Nuclear-Plastid Incompatibility

In a few published studies, the molecular genetic bases of incompatibility of the nuclear genome and plastids has been addressed. In cybrids of Solanacea and in interspecific crosses of *Oenothera,* the plastid factors were postulated while the nuclear factors remained unknown. In pea, conflicting factors of both plastid and nuclear origin were nominated. In wheat, molecular genetic dissection of the nuclear factor of cytonuclear compatibility received little attention, although it is prominent in that it demonstrates the power of genetic analysis. It remains unknown whether the involved cytoplasmic factors originate from plastids, mitochondria, or both. 

### 4.1. Molecular Genetic Analysis of Nuclear-Plastid Incompatibility in Solanaceae

One of bright examples of nuclear-plastid incompatibility is represented by cybrids of Solanaceae plants obtained by a protoplast fusion and regenerated in cell culture. For example, plants with the nuclear genome of belladonna (*Atropa belladonna* L.) and chloroplasts of tobacco (*Nicotiana tabacum* L.) completely lack chlorophyll pigmentation [26]. A very elegant study of the molecular basis of incompatibility in such plants employed a genetic approach [82]. Cell cultures with an incompatible combination of nucleus (*A. belladonna*) and chloroplasts (*N. tabacum*) were treated with nitrosomethylurea to induce mutations restoring normal chlorophyll pigmentation. Three independent mutations were obtained that were associated with the outgrowth of green shoots. Chloroplast transfer between cell cultures with intact nuclei showed that these mutations occurred in the tobacco plastid genome. 

Earlier, the plastid genome of belladonna was sequenced, and sites potentially involved in the conflict were revealed [83]. These regions of the tobacco plastid genome were sequenced in the mutants with a restored compatibility. In the three mutants mentioned above, specific nucleotide substitutions were found, as well as a substitution common for all mutants. The latter substitution in the gene *atpA,* coding for alpha subunit of the ATPase, converted 264-th codon, CCC (proline), into CTC (leucine). Normally in tobacco, the cytosine base which had undergone the mutation is edited at the mRNA level and is turned into uracil, thus, converting the CCC-Pro codon into the CUC-Leu. In contrast, in belladonna, leucine is encoded by the CTC codon [83]. Thus, the identified mutation did not change the amino acid sequence of the AtpA protein but abolished the need for RNA editing. RNA-editing factors are known to be encoded in the nucleus [84,85], and apparently belladonna genome lacks the factor which specifically edits the cytosine in the 264-th codon of the *atpA* gene, resulting in the amino acid replacement of leucine by proline L264P [82]. 

### 4.2. Molecular Genetic Analysis of Nuclear-Plastid Incompatibility in Evening Primroses

As mentioned earlier, in *Oenothera*, three types (A, B, and C) of nuclear genomes and five types (I to V) of plastid genomes (plastomes) are described that produce different compatible and incompatible combinations [14,16]. An attempt was made to establish the molecular determinants in these plastid genomes involved in nuclear-cytoplasm incompatibility. 

The five types of the plastid genomes were sequenced [86], and variants of the nucleotide sequences were considered in relation to the patterns of compatibility and incompatibility of their carriers. If a DNA sequence variant was found in all plastomes compatible with a certain genome (for example, A), while all incompatible plastomes carried another structural element, such locus was considered as a candidate [87]. Unfortunately, interspecific variability of the plastomes turned out to be very high, and pairwise comparisons revealed thousands of differences. The comparison of compatible and incompatible plastid genomes allowed 82 candidates to be chosen, 19 of which corresponded to protein-coding loci, and 63 of which corresponded to promoter regions in intergenic spacers. Out of the promoter region-specific loci, 38 were predicted to be binding sites for PEP (plastid-encoded polymerase) RNA-polymerase and 25 predicted to be binding sites for NEP (nuclear-encoded polymerase). Participation of some of the identified genes, for example those coding for subunits of NADH dehydrogenase, in incompatibility was considered not likely, since their disruption in the tobacco plastid genome did not cause notable phenotypic change [88]; although this conclusion was argued against [89]. To narrow the candidate list, the authors [87] applied a series of rather arbitrary criteria, such as too high or too low variability. They also discarded the candidates associated with the differences not affecting functional domains or biochemical properties of the encoded proteins. 

Plants of one incompatible combination were deficient in chlorophyll pigmentation and displayed a reduced activity of the photosystem II. Therefore, the authors suggested that the chloroplast factor of incompatibility of the plastome I with the genome AB (heterozygote for A and B) resided in the intergenic spacer *clpP-psbB.* This intergenic spacer contains a 148-bp deletion which affects the promoter for the NEP polymerase of the *clpP* gene and a predicted promoter element for the PEP polymerase of the *psbB* gene located in two different DNA strands [87]. The nuclear factor of incompatibility remains unknown.

### 4.3. Molecular Genetic Analysis of Nuclear-Plastid Incompatibility in Peas

In order to reveal the plastid participant of the nuclear-plastid conflict in pea, five chloroplast genomes were sequenced that originated from the accessions contrasting in compatibility with the nuclear genome of cultivated pea. The study focused on a search for the plastid genes interacting with the nuclear *Scs1*, since its effect was more pronounced and did not depend on the other nuclear loci, unlike *Scs2* (see above). 

The features of the primary structure that were similar in incompatible plastid genomes and different in compatible genomes were regarded as candidates responsible for nuclear-plastid compatibility. Thirty-seven such candidates were found in 14 non-coding regions and seven protein-coding loci. Changes of the protein primary structure were considered a more likely cause for incompatibility, and the range of candidates was narrowed to four protein-coding loci (*accD*, *rpoB*, *ycf1,* and *ycf2*). 

Nuclear-cytoplasmic incompatibility is expected if nucleus- and plastid-encoded subunits of multimeric complexes poorly match each other [13]. In such a case, the most probable plastid candidate gene should have a nuclear-encoded counterpart mapped to the region of linkage group III where *Scs1* was located. On the basis of the synteny of legume genomes [90], a search was made in the annotated genome of *Medicago truncatula* of the loci encoding proteins participating in formation of complexes with the products of selected plastid candidate genes. The genetic interval where *Scs1* was mapped corresponded to a stretch of 1.2 Mb of *M*. *truncatula* chromosome 3 containing 166 annotated gene products, one of which was “biotin carboxyl carrier protein of acetyl-CoA carboxylase” (GeneID:11410363). Thus, the plastid *accD* gene, coding for the beta subunit of carboxyltransferase of plastid multimeric acetyl-CoA carboxylase, was identified as a candidate gene involved in the nuclear-plastid incompatibility, and, for the first time, both nuclear and plastid participants were nominated [91]. The proposed model of incompatibility implies a failure of the protein–protein interaction in a multimeric enzyme complex of acetyl-CoA carboxylase. This model was further substantiated by genetic mapping of *Scs2* to a region which, in *M*. *truncatula.,* harbors the *AccA* locus coding for alpha subunit of carboxyltransferase of acetyl-CoA carboxylase [91]. Allelic polymorphism in the candidate genes was extensively studied [92].

### 4.4. Molecular Genetic Analysis of Nuclear-Plastid Incompatibility in Cereals

Detailed genetic mapping of nucleus-cytoplasm compatibility (*scs*) loci on wheat chromosomes 1A and 1D (see above) facilitated the identification of the nuclear candidate gene responsible for the interaction with the cytoplasm. Bulked segregant analysis was applied, where the negative bulk contained DNA of the lines with a radiation-induced deletion of *scs ^ae^* allele, while the positive bulk contained DNA of the lines with the functional *scs ^ae^* [93]. These bulks were sequenced, and the detected SNPs allowed identifying markers which were then used to genotype a population of 644 radiation hybrid lines. 

As a result, the genetic position of *scs* was further refined, and the physical map of a BAC clone of *Ae*. *taushii,* spanning the region of interest and comprising about 500 Kb, was scrutinized. It turned out that the ORF representing a putative *rhomboid* gene was tagged by Xndsu297, the only marker cosegregating with the *scs ^ae^* phenotype. The *rhomboid* gene was sequenced from different sources characterized by functional and non-functional alleles of *scs,* and it was established that, in all non-functional allelic variants, the arginine residue was replaced with lysine [93]. 

Rhomboids constitute a family of intramembrane cleaving proteases that are key regulators of critical cellular processes involved in the maintenance of the structural and functional integrity of mitochondria and plastids [94], although their link with the developmental processes is still missing [95]. A possible mechanism of the effect of a protease on an organelle function is exemplified by an improper N-terminus processing of light harvesting proteins in alien henbane (*Hyoscyamus niger* L.) chloroplasts in cybrids with tobacco nucleus [96].

## 5. Concluding Remarks

The evolutionary significance of cytonuclear conflicts has been widely recognized [8,11,13]. Consequently, a question arises, if there exist certain genes or gene types predisposed to cause hybrid incompatibilities, thus contributing to postzygotic reproductive isolation. All the studies of plastid-nuclear incompatibility, reviewed in this review, refer to different genetic loci and may be classified according to the pathways of interaction between nuclear-encoded and organelle-encoded products as protein-RNA (Solanacea cybrids), protein-DNA (*Oenothera*, presumable), or protein-protein (pea, possibly wheat). The evolutionary significance of protein–protein interactions was substantiated by a number of studies. There are some plastid genes characterized by accelerated rates of nucleotide changes in some plant lineages, for example, *clpP* and *accD* ([97] accompanied by an increase in the rate of evolution of the corresponding nuclear-encoded subunits, which was supposed to reflect antagonistic coevolution between plastids and nucleus [97]. The same situation of rapid coevolution between plastid-encoded RNA polymerase and nuclear-encoded sigma-factors [98] and plastid-and nuclear-encoded subunits of ribosomes [99] was observed in Geraniaceae. In cross-hybridizations, such rapidly evolving genes with high probability would create poorly compatible combinations and reduce the fitness of their carriers, contributing to reproductive isolation, and therefore could be referred to as “speciation genes” [9].

Regardless of the nature of cytonuclear incompatibility, the knowledge of its genetic and molecular genetic basis can help overcome the restrictions it imposes, and design breeding programs aimed at employment of natural biodiversity, to improve existing crops.

## Figures and Tables

**Table 1:** Experimental studies to elucidate the genetic basis of nuclear-plastid incompatibility.

Plant Genus	Type of Cross or Line	Experimental Approach	Observations	Conclusion	Ref
*Medicago*	Backcross of F_1_ to one of the parental lines in reciprocal directions	Analysis of segregation ratio for chlorophyll pigmentation	1:1 segregation	Monogenic inheritance	[51]
*Acacia*	F_2_ from interspecific cross	Analysis of segregation ratio for chlorophyll pigmentation	Lack of clear segregation ratios	Inconclusive	[55]
	Backcross of F_1_ to parental species-donor of cytoplasm	Analysis of segregation ratio for chlorophyll pigmentation	Lack of clear segregation ratios, high frequency of mosaics	Hybrid chlorosis assigned to nuclear-nuclear interactions	[55]
*Oenothera*	F_2_ from interspecific crosses	Cosegregation of visible markers and chlorophyll pigmentation	Linkage of two visible markers to chlorophyll deficiency	Assignment of incompatibility loci to chromosome arms marked by visible markers	[56]
	Backcross of interspecies F_1_ hybrid to both parental species on different cytoplasm backgrounds	Cytological analysis and association of certain chromosomes with chlorophyll deficiency	Degree of chlorosis related to the number of foreign chromosomes	Nuclear factors of incompatibility scattered all over genome	[59]
	F_2_ from interspecific cross	Cosegregation of molecular markers and chlorophyll pigmentation	Linkage of some molecular markers to chlorophyll deficiency	Segregation of at least two nuclear factors of incompatibility; one on chromosome 4 and the other–on chromosome 7	[60]
*Zantedeschia*	F_2_ from interspecific cross and backcrosses	Analysis of segregation ratio for chlorophyll pigmentation	Lack of clear segregation ratios	Inconclusive	[23]
*Rhododendron*	F_1_ from crosses of species of different ploidy	Correspondence of genome constitution and chlorophyll pigmentation	dependence of chlorophyll deficiency on the dosage of maternal nuclear genome	Presence of dominant dosage-dependent alleles	[63]
*Triticum* and *Aegilops*	Alloplasmic lines on background of *Ae. taushii* cytoplasm	Substitution of individual chromosomes from D genome by homoeologous chromosomes of A and B genomes	Invariable presence of 1D chromosome in compatible combinations	Presence of compatibility factors on 1D chromosome	[45]
	Backcrosses of F_1_ from cross of alloplasmic lines on background of *Ae. taushii* cytoplasm with incompatible or partially compatible wheat species to incompatible(partially compatible) parental line	Analysis of segregation ratios for chlorophyll variegation and plant vigor	Segregation ratios corresponding to two-gene control	Two types of loci involved in compatibility with *Ae. taushii* cytoplasm, one interacting with chloroplasts and a second related to plant vigor	[45]
	Substitution lines with compatibility locus introgressed from *T. timopheevii* on background of *Ae. taushii* cytoplasm	Southern blot analysis	Disappearance of hybridization signal along with 1A chromosome substitution	Presence of compatibility factors on 1A chromosome from *T. timopheevii*	[66]
	Test crosses of alloplasmic lines on background of *Ae. taushii* cytoplasm with *T. timopheevii*	Analysis of segregation ratios for seed viability	Segregation ratios corresponding to two compatible alleles	Presence of compatibility factors on 1G chromosome from *T. timopheevii*	[67]
	Test cross of an alloplasmic line on background of *Ae*. *longissima* cytoplasm with near-isogenic line heterozygous for compatibility loci	Cosegregation of RFLP markers and traits related to plant vigor and seed viability, Southern blot analysis	Southern blot hybridization using DNA markers linked to compatibility loci	Positioning of one compatibility locus in the distal part of the chromosome arm 1BS, and the second–in the chromosome arm 1AL close to the centromere	[64]
	F_2_ from a cross of homozygous lines, one parent with introgressed compatibility locus from *T. timopheevii*	Genotyping of F_2_ progenies by an additional test cross	Cosegregation of some molecular markers with seed viability	Compatibility locus mapped on chromosome 1A with bordering markers at a distance of 2.3 and 0.6 cM	[46]
	F_1_ from a cross of irradiated alloplasmic line on background of *Ae*. *longissima* cytoplasm added with 1D chromosome with euplasmic line (radiation hybrids)	Presence/absence of molecular markers specific for 1D	Finding of markers constantly present in radiation hybrids compatible with *Ae. longissima* cytoplasm	Molecular markers covering a stretch of about 8.3 Mb denoting position of a compatibility locus on chromosome arm 1DL	[75]
*Pisum*	Population of recombinant inbred lines	Genotyping of lines from mapping population by additional crosses to a testerline with incompatible cytoplasm	Cosegregation of some visible and molecular markers with compatibility/incompatibility	Segregation of two nuclear loci for incompatibility, one on linkage group III and a second on linkage group V	[78,79]
